# Predicting escitalopram monotherapy response in depression: The role of anterior cingulate cortex

**DOI:** 10.1002/hbm.24872

**Published:** 2019-11-22

**Authors:** Shui Tian, Yurong Sun, Junneng Shao, Siqi Zhang, Zhaoqi Mo, Xiaoxue Liu, Qiang Wang, Li Wang, Peng Zhao, Mohammad Ridwan Chattun, Zhijian Yao, Tianmei Si, Qing Lu

**Affiliations:** ^1^ School of Biological Sciences and Medical Engineering Southeast University Nanjing China; ^2^ Child Development and Learning Science Key Laboratory of Ministry of Education Nanjing China; ^3^ Department of Psychiatry Affiliated Nanjing Brain Hospital, Nanjing Medical University Nanjing China; ^4^ Nanjing Brain Hospital, Medical School of Nanjing University Nanjing China; ^5^ Peking University Institute of Mental Health & Sixth Hospital Beijing China; ^6^ National Clinical Research Center for Mental Disorder & The Key Laboratory of Mental Health, Ministry of Health Ministry of Health (Peking University) Beijing China; ^7^ Department of Medical Psychology Affiliated Nanjing Drum Tower Hospital, Medical School of Nanjing University Nanjing China

**Keywords:** anterior cingulate cortex (ACC), default mode (DMN) subnetwork, escitalopram, modular structure, support vector machine (SVM)

## Abstract

Neuroimaging biomarkers of treatment efficacy can be used to guide personalized treatment in major depressive disorder (MDD). Escitalopram is recommended as first‐line therapy for MDD and severe depression. An interesting hypothesis suggests that the reconfiguration of dynamic brain networks might provide important insights into antidepressant mechanisms. The present study assesses whether the spatiotemporal modulation across functional brain networks could serve as a predictor of effective antidepressant treatment with escitalopram. A total of 106 first‐episode, drug‐naïve patients and 109 healthy controls from three different multicenters underwent resting‐state functional magnetic resonance imaging. Patients were considered as responders if they had a reduction of at least 50% in Hamilton Rating Scale for Depression scores at endpoint (>2 weeks). Multilayer modularity framework was applied on the whole brain to construct features in relation to network dynamic characters that were used for multivariate pattern analysis. Linear soft‐threshold support vector machine models were used to separate responders from nonresponders. The permutation tests demonstrated the robustness of discrimination performances. The discriminative regions formed a spatially distributed pattern with anterior cingulate cortex (ACC) as the hub in the default mode subnetwork. Interestingly, a significantly larger module allegiance of ACC was also found in treatment responders compared to nonresponders, suggesting high interactivities of ACC to other regions may be beneficial for the recovery after treatment. Consistent results across multicenters confirmed that ACC could serve as a predictor of escitalopram monotherapy treatment outcome, implying strong likelihood of replication in the future.

## 1 INTRODUCTION

Major depressive disorder (MDD) affects approximately 216 million individuals during their lifetime (Katikireddi, 2017) and constitutes the second leading course of years lived with disability worldwide (Global Burden of Disease Study, 2015). Antidepressants are commonly used to treat MDD, especially for those with moderate to severe depression.

Escitalopram is an allosteric selective serotonin reuptake inhibitor (SSRI) that exhibits a superior efficacy and tolerability compared to other SSRIs in several randomized controlled trials (Cipriani et al., 2009; Sanchez, Reines, & Montgomery, 2014). On average, it takes 8–12 weeks for evaluating the treatment efficiency of individuals with MDD. The response rates of initially administered antidepressants normally ranges 50–75% (Listed, 2000). However, numerous studies have suggested that treatment with antidepressants may lead to a response rate of only 30–40%, resulting in a large number of patients with continued “alternative lifestyle” (Crane et al., 2017; Holtzheimer & Mayberg, 2011; Trivedi et al., 2005; Williams, 2017). In cases where patients do not respond to one SSRI, treatment can either be switched to another antidepressant (Whooley & Simon, 2000) or to an atypical antidepressant, which could conservatively be more effective than SSRIs (Papakostas, Thase, Fava, Nelson, & Shelton, 2007; H. R. Wang et al., 2015). However, it is noteworthy that every medication trial takes weeks, prolonging the disability. In the meantime, frustration due to delays in remission increases the risk of adverse outcomes such as suicide. If treatment response of individuals could be predicted, clinical intervention would be optimized.

Convergent neuroimaging biomarker studies focused on the main underlying pathophysiologic processes in depression provided a better understanding of treatment mechanism and a possibility for the development of personalized treatment strategies (Phillips et al., 2015). A systematic review (Dichter, Gibbs, & Smoski, 2015) linking resting‐state functional magnetic resonance imaging (fMRI) with treatment response reported an increase in functional connectivity (FC) between frontal and limbic regions in responders. A higher connectivity within cognitive control network (CCN) and a negative correlation of the anterior cingulate cortex (ACC) with the subcallosal cortex (Kozel et al., 2011) were reported to be able to identify the preferred treatments for individuals with MDD. Cerebellar connectivity (Guo et al., 2013) and interaction among visual recognition circuits (L.‐J. Wang, Kuang, Xu, Lei, & Yang, 2014) were also suggested to be potential predictors. In addition, intra/inter hyperconnectivity in relation to default mode network (DMN) has been identified in treatment resistant depression compared to treatment sensitive depression. This potentially suggested that lower DMN related connectivity is a feasible predictor of effective antidepressant medications (Ma et al., 2012). Beata and others (Godlewska et al., 2018) have also shown that pretreatment pgACC activity is predictive of response to escitalopram after 6 weeks. FC of subcallosal cingulate cortex could identify individuals' treatment outcome to cognitive behavioral therapy (CBT) and antidepressant medicine (Dunlop et al., 2017).

However, it is important to note that most of these studies that examined the antidepressant effects on the connectivity or circuit level rarely explored the underlying dynamic characteristics. Recent studies have revealed brain modular‐level abnormalities in MDD patients (Tao et al., 2013; Tian et al., 2019; Zheng et al., 2017), suggesting that modular‐related properties may be more sensitive than regional properties in reflecting brain alterations. In addition, the dynamic brain network reconfiguration of schizophrenic patients with antipsychotic medication was reported to have a significant “network hyper‐flexibility” (Braun et al., 2015). Therefore, the topic is of particular interest in light of growing understanding that MDD is not only associated with abnormalities of a single or independent brain region, but also with systems level dysfunction affecting discrete functionally integrated neural circuits. An advanced study should be able to extend the conventional methodological framework for a better understanding of the discrepancy concerning mechanisms of MDD.

Truly, the two complementary principles of large‐scale brain networks namely, functional segregation and dynamic integration, became of considerable interest. However, it is challenging to take them into account simultaneously. In 2010, Mucha and Onnela (2010) developed a generalized framework to study the community structure of time‐dependent, multiscale, and multiplex networks. It has been introduced on neuroimaging data for better understanding our brain (Bassett et al., 2011; Braun et al., 2015, 2016). After modularizing the multislice community structure over time, the node flexibility as the network parameters can be used to characterize the dynamic community structure. The node flexibility was defined as the number of times that node changed its modular allegiance normalized by the total number of changes that were possible across the scanning time (Bassett et al., 2011). It was suggested that successful brain function might partly depend on a set of regions whose allegiance to putative functional modules is flexible through time to smooth the function transition (Hermundstad et al., 2014). In this study, we try to find a personalized escitalopram monotherapy treatment marker for treatment prediction, with an interesting hypothesis that reconfiguration of dynamic brain network might provide important insights into the depressive disorder and also be potential for effective antidepressant treatment prediction.

## 2 MATERIALS AND METHODS

### 2.1 Study population

In this cohort study, we examined a total of 106 first‐episode, drug‐naïve patients from three different hospitals. Thirty‐five Han Chinese depressive inpatients were recruited from Nanjing Brain Hospital between May 2010 and October 2017 (Sample 1). Thirty‐six outpatients were enrolled from the Nanjing Drum Tower Hospital between September 2014 and October 2017 (Sample 2). Thirty‐six Chinese outpatients were recruited from the Peking University Institute of Mental Health between May 2010 and July 2016 (Sample 3). In addition, a total of 109 healthy controls from these centers were recruited for comparing analyses.

The diagnosis of MDD was validated using the Mini‐International Neuropsychiatric Interview by at least two psychiatric physicians (Sheehan et al., 1998). Patients were also assessed with Hamilton Depression Rating Scale 17‐Item (HDRS‐17) (Hamilton, 1967) and Hamilton Anxiety Scale (HAM‐A) (Maier, Buller, Philipp, & Heuser, 1988).

### 2.2 Inclusion and exclusion criteria

First‐episode patients suffering from an acute episode of depression, with a HDRS‐17 total score > 17, were included. The patients needed to have experienced symptoms of depression for ≤24 months but ≥1 month. None of the subjects have repetitive transcranial magnetic stimulation (rTMS), CBT, or other forms of psychotherapy during the study period. Subjects with a history of alcohol and substance abuse were also excluded. Patients that had comorbidity with other Axis I or Axis II disorders psychiatric illnesses were ruled out. Pregnant patients and those who are unable to undergo a MRI scans were excepted. The patients who changed medications due to serious side effects were not considered for further analysis. Similar exclusion criteria were made for controls.

In this study, responders were conventionally defined as those having a reduction of at least 50% in HDRS‐17 scores at endpoint (≤8 weeks). These patients showed a clinical improvement to escitalopram treatment, with a more than 20% decrease from the baseline HDRS‐17 scores at 2 weeks. Patients who changed medications or received electroconvulsive therapy due to poor improvement (a reduction of less than 20% in HDRS‐17 scores) after at least 2 weeks escitalopram administration or had a reduction of less than 50% in HDRS‐17 scores at endpoint were defined as nonresponders. One participant who had large head motion from Site 1 and one who had cerebral cysts from Site 2 were discarded from further neuroimaging analysis.

### 2.3 Escitalopram administration

All participants underwent a baseline functional MRI scan, following which they consented to commence escitalopram treatment at a dose of 10 mg/day. Afterward, the dose for each individual was determined by each patient's response and tolerance. After 7 days, patients had their escitalopram dosage increased during the study period. The final escitalopram dosage in the three multicenters (Nanjing Brian Hospital/Drum Tower Hospital/Peking University Institute of Mental Health) were 20 mg/day (*n* = 15/4/11), 15 mg/day (*n* = 15/20/19), and 10 mg/day (*n* = 4/10/6). The average dose (±*SD*) at the endpoint was 16.6 ± 3.4/14.1 ± 3.1/15.7 ± 3.4 mg.

### 2.4 MRI data acquisitions

Participants were not allowed to carry any piece of metal in the magnetically shielded room. They were instructed to keep their eyes closed, not fall asleep and minimize movement. Head motion was confined to less than 1.5 mm in any direction. No subjects were reported to fall asleep during the scanning.

Data from the first and second cohorts were collected by a 3.0T Siemens MRI Scanner (Siemens Medical solutions, Germany) equipped with a 12‐channel neurovascular array coil. Resting‐state functional images were recorded using echo‐planar imaging sequence, yielding whole‐brain coverage in all participants. (repetition time [TR]/echo time [TE] = 3,000 ms/40 ms; flip angle [FA] = 90°; matrix = 64 × 64; thickness/gap = 4.0 mm/0 mm; slice number = 32). The recording session lasted 6 min 45 s. The T1‐weighted anatomic images were obtained by gradient‐echo sequence (TR/TE/FA = 1,900 ms/2.48 ms/9°).

Images from the cohort Sample 3 were also acquired by a 3.0T Siemens MRI Scanner with the following parameters: TR/TE = 2,000 ms/30 ms, FA = 90°, matrix = 64 × 64, thickness/gap = 4.0 mm/0.8 mm, number of slices = 30, and lasting scan time = 7 min. The parameters for the T1‐weighted anatomic images were TR/TE/FA = 2,300 ms/3.01 ms/9°.

### 2.5 Image preprocessing

The first 10 functional volumes were deleted for signal equilibrium and to allow the participants' adaptation to the machine noise. Data preprocessing was handled via the Data Processing Assistant for Resting‐State fMRI (DPARSF) (Yan & Zang, 2010) and the Statistical Parametric Mapping software (SPM8; http://www.fil.ion.ucl.ac.uk/spm). Functional images underwent slice‐timing and head‐motion correction on a participant‐level confound regression model which combined six movement estimates and three physiological time series and censored volumes preceding any movement (frame‐wise displacement) greater than 0.3 mm (Ciric et al., 2017; Drysdake et al., 2017). The functional images were then normalized into the Montreal Neurological Institute (MNI) space and the parameter was set as 3 × 3 × 3 mm^3^. After that, functional images were smoothed with 6‐mm full‐width at half‐maximum Gaussian kernel and band‐pass filtered (0.01–0.08 Hz) to reduce the effects of low‐frequency drift and high‐frequency noise.

### 2.6 Data analyses

The signals of each region were extracted via a sphere with a 6 mm radius based on a template in a previously published article by Allen et al. (2014). A table for the definition of these regions by node location and MNI coordinates has been added in the Table S2. The whole brain dynamic FC was determined using pair‐wise Pearson correlation between 95 regions of interest with sliding window width = 45 TRs, sliding step = 1 TRs. The resulting correlation matrices of each subject were partitioned into time‐dependent communities using a multilayer community detection algorithm (approach details can be referred to the Supporting Information and our previous work; Shao et al., 2019; Tian et al., 2019). This framework embodies the notion of segregation and integration, as the presence of modules/communities reflects the balanced interactive between intra‐ and interclusters. Hence, community detection methods enable the investigation of the interplay between functional segregation and integration of brain, together with numerous dynamic characteristics that other standard graph‐theoretical measures such as degree, rich club, and small‐word property cannot offer.

Subsequently, module allegiance (MA) matrices, demonstrated the probability of two nodes being assigned to the same module/communities, were utilized to analyze the dynamic integration across large‐scale networks. We obtained MA matrices by making contingency tables (1 if two regions were assigned in the same community; and 0 otherwise) and averaging all the variables such as time and iterations. The larger the MA presented, the stronger connection the pair of nodes possessed over the time domain. As was suggested via the findings of Bassett, Yang, Wymbs, and Grafton (2015), the intercluster interaction can be better delineated by the MA matrices than done by FC matrices.

In order to capture the temporal variability for nodes in MA, we calculated a time‐dependent system flexibility vector *F*. After normalized by the total number of time windows, each element *F*_*i*_ indicates the times that a node *i* changes its modules between two consecutive time windows in personal level. In this way, the representative flexibility resulted in a total of 95 features (one for each region) for predicting individual treatment outcome.

### 2.7 Multivariate pattern analysis

Multivariate pattern analysis (MVPA) was a well‐suitable method and was conducted to discern slight differences underlying the *F* values (Mohr, Wolfensteller, & Ruge, 2017). The input features for the MVPA were constructed by identifying subsets of data that are most relevant to the treatment outcome using minimum redundancy maximum relevance (mRMR) (Peng, Long, & Ding, 2005). As a popular approach over neuroimaging studies, the linear soft‐threshold support vector machine (SVM) model (linear kernel, soft margin *C* = 1) (Schrouff et al., 2013) was used to separate responders from nonresponders.

The model accuracy was tested using leaving‐*one*‐out cross validation (LOOCV) (Cawley & Talbot; Varoquaux et al., 2016). In this procedure, each subject was left out once and used to test the prediction model trained on the other subjects. In addition, we used a discriminative mapping approach to visualize the relative contribution of the different brain regions for the classifier decision. To eliminate site effects, the leave‐*one‐group*‐out analyses were also applied.

### 2.8 Permutation test

The statistical significance of the accuracy and weight was tested by randomly permuting the labels of the training samples with the derived models. This process was repeated 1,000 times to determine the null‐distributions of accuracies/weights. The *p* value of accuracy/weight was calculated from the permutation procedure that had a larger accuracy/weight compared to the null model. The value and significance of the weights provided an indication of the relative importance of the respective features for classification and prediction. It should be noted, however, that these values must be interpreted with care. The weights indicated the direction and quantified the contribution of each node to classifier decision rather than unfolded the difference between classes.

### 2.9 Statistical analysis

The interaction among meaningful discriminative regions was compared between responders and nonresponders according to two‐sample two‐tailed *t* tests. In order to account for multiple comparisons and counteract the likelihood of false positives, false‐discovery rate (FDR) correction was applied (Storey, 2003). The statistically significant difference was set at *p* < .05.

## 3 RESULTS

### 3.1 Demographic and clinical characteristics

The demographic and clinical characteristics of the subjects were summarized in Table 1. There were no statistical differences in the demographic variables between responder and nonresponder individuals within the three separate cohorts, respectively, including age, gender, years of schooling, and symptom scores. There were no statistical differences in the demographic variables between depressive individuals and healthy controls as well (see Table S1). The changes of depression severity were scatter plotted in Figure S1.

**Table 1:** The demographic characteristics of the multicenter

	Responders	Nonresponders	*p*
Numbers of subjects	19/17/20	15/19/16	—
Age (years)	33.74 ± 11.97/31.88 ± 7.43/35.80 ± 10.34	33.93 ± 9.39/29.37 ± 4.60/31.47 ± 9.60	.959/.268/.199^a^
Education (years)	12.31 ± 1.89/14.10 ± 2.93/13.69 ± 2.68	12.53 ± 3.02/14.91 ± 4.26/12.84 ± 2.69	.799/.866/.876^a^
Length of depressive episode (months)	5.640 ± 3.75/5.04 ± 5.22/‐	5.50 ± 6.65/5.62 ± 4.11/‐	.942/.764^a^/‐
Age of index episode (years)	33.27 ± 10.56/31.46 ± 6.48/‐	33.47 ± 7.69/28.91 ± 3.80/‐	.938/.374^a^/‐
Gender (male /female)	9M10F/8M9F/10M10F	11M4F/10M9F/7M9F	.097/.600/.709^b^
Handedness (left or right)	0L19R/0L17R/0L20R	0L15R/0L19R/0L16R	>.999^b^
Total HDRS score	23.95 ± 5.09/25.18 ± 5.95/27.5 ± 3.8	24.27 ± 4.96/25.47 ± 5.45/‐	.855/.918^a^/‐
Anxiety	6.00 ± 2.57/6.80 ± 2.37/‐	5.90 ± 1.76/6.78 ± 1.81/‐	.919/.988^a^/‐
Weight loss	1.00 ± 0.94/0.87 ± 0.91/‐	1.00 ± 0.89/0.94 ± 0.97/‐	.912/.807^a^/‐
Cognitive disturbance	4.17 ± 2.85/4.73 ± 1.71/‐	3.81 ± 2.14/4.37 ± 1.61/‐	.722/.527^a^/‐
Retardation	7.41 ± 1.06/8.60 ± 1.72/‐	8.27 ± 1.73/8.16 ± 1.38/‐	.115/.413^a^/‐
Sleep disturbance	4.41 ± 1.73/3.13 ± 1.45/‐	4.27 ± 1.90/3.68 ± 1.66/‐	.847/.320^a^/‐

*Note*: Values shown are listed as Nanjing Brain Hospital/Nanjing Drum Tower Hospital/Peking University Institute of Mental Health (mean ± *SD*).

Abbreviation: HDRS, Hamilton rating scale for depression.

a Two‐sample *t* test;

b Pearson Chi‐square test.

### 3.2 Dynamic network module

**Figure 1:**
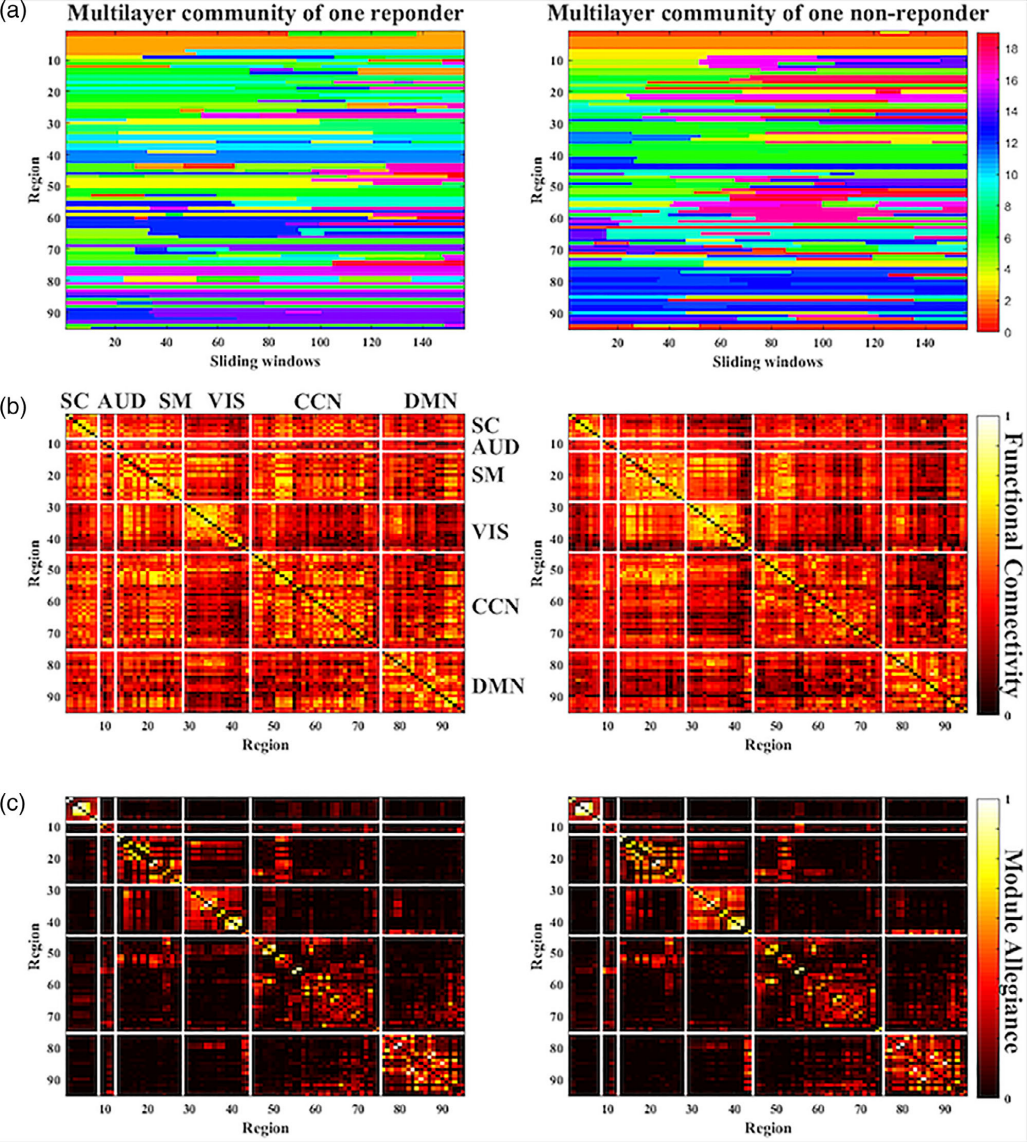
Dynamic modular structure and functional connectivity. (a) The community assignment of regions along the time windows of one random responder and nonresponder. The horizontal axis represents the continuous time‐windows, while the vertical axis represents the regions of interest from Allen et al. (2014). A region possibly belongs to the same module across a series of continuous time windows until it gets transferred to another module in the succeeding instance. The colors depict the respective community assignments. (b) The functional connectivity matrices show the averaged Pearson correlation between regions of interest for responders and nonresponders. (c) The averaged module allegiance matrices for the two subject groups illustrate the probability of areas being in the same community across time windows and subjects. The white line showed the predefined “functional networks”

Dynamic modules, in this case, represent the groups of mutually correlated brain regions, which are weakly connected to the rest of network along time. We investigated modular organization across the brain networks in both responders and nonresponders using multilayer modularity framework. The multilayer community assignment of one randomly selected responder and nonresponder were showed in Figure 1a. Six networks were transiently integrated and segmented and thereafter, rearranged into special communities across the intrinsic networks. The results of averaged FC matrices and averaged MA matrices within each group were compared. The intrinsic functional architecture was better delineated via MA matrices. Regions within the DMN were more likely to be assigned into the same modules while the CCN integrated with other intrinsic networks (Figure 1b,c). Similar results for the samples in second and third sites were showed in Figures S2 and S3.

### 3.3 SVM predictors for effective escitalopram treatment

**Figure 2:**
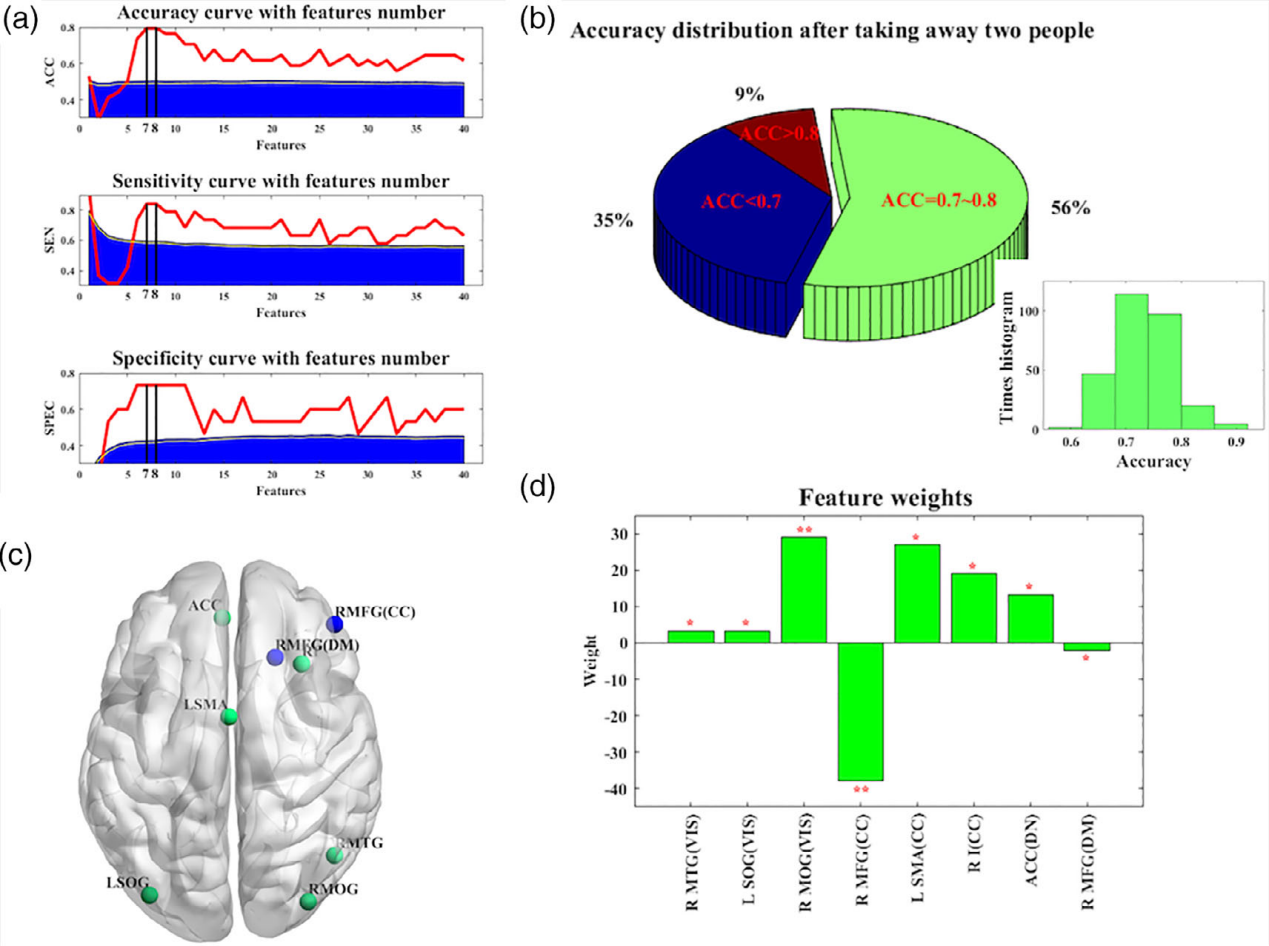
Performance of SVM classification and mapping of feature weights. (a) The red line shows the performance of SVM models using real data and the blue area represents the performance required for statistical significance (*p* < .001), derived from the null distribution. (b) The accuracy of SVM models was tested for robustness performance. The lower histogram shows the distribution of accuracy and the pie chart depicts most of possibilities concentrated on the accuracy of 70–80%. (c) The summary of features with high discriminative power. Relevant brain regions were mapped and color‐coded by weight directions. Positive weights were green, negative ones were blue. (d) The selection frequency of features' physiological parameters. For each plot, the permutation tests were applied 1,000 times. A single asterisk indicated *p* < .01, double asterisks indicated *p* < .005. ACC, bilateral anterior cingulate cortex; CC, cognitive control network; DM, default mode network; L SMA, left supplementary motor area; L SOG, left superior occipital gyrus; R I, right insula; R MFG, right middle frontal gyrus; R MOG, right middle occipital gyrus; R MTG, right middle temporal gyrus; SVM, support vector machine; VIS, visual network

There was no significant difference in flexibility between the responders and nonresponders (see details in Figure S4). Feature wrapping approach of mRMR selected the related flexibility features while SVM models were applied to differentiate responders from the other patients. The real LOOCV accuracy was 79.41%, while sensitivity and specificity were 84.21% and 73.33%, respectively (see Figure 2a).

Sample balance is really an important point in modeling, especially for small sample cases (Scheinost et al., 2019). In order to retest the robustness of our model, one participant from each of the two groups was taken away while the above framework was reapplied to the remaining subjects. Then, we cross‐checked all the 285 (19*15) sample combination possibilities. Figure 2b illustrates the distribution of optimum classification results over difference sample combination, with a peak possibility concentrated around the range of 70–80%. These leave‐*two*‐out results implied that the discrimination performance was robust and not just a mere coincidence arisen by limited samples.

### 3.4 Map of discriminating regions

The first eight discriminative regions of the optimal classier were displayed on Figure 2c,d. This map contained positive (green) and negative (blue) weights, involved in decision‐making. Actually, these regions are distributed across different networks in both hemispheres (Figure 2c). Furthermore, permutation analyses demonstrated the significantly discriminative regions compared to null models, including the right middle temporal gyrus (R MTG, *p* = .035), right middle occipital gurus (R MOG, *p* = .037), left superior occipital gyrus (L SOG, *p* < .001), right middle frontal gyrus (R MOG, *p* = .001), left supplementary motor area (L SMA, *p* = .003), right insula (R I, *p* = .008), bilateral ACC(*p* = .011), and right middle frontal gyrus (R MFG, *p* = .049) (see Figure 2d).

### 3.5 MA of responders and nonresponders

**Figure 3:**
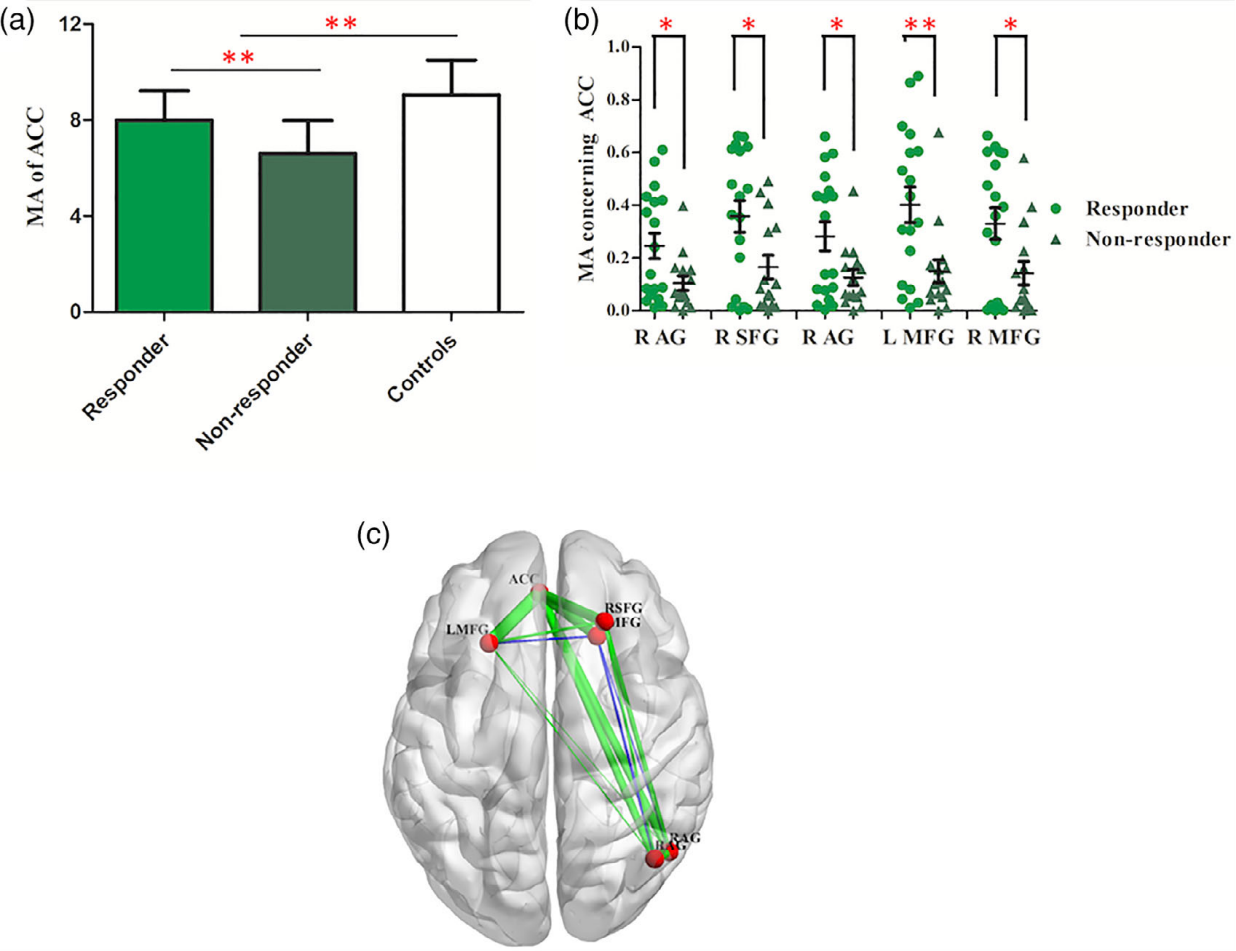
Module allegiance matrices of the key regions relating to the ACC. (a) The histogram illustrated the MA between ACC and other brain regions in responders, nonresponders, and healthy controls. Patients with depression showed lower MA than healthy controls while responders possessed larger one than nonresponders. Bars indicate mean values, and whiskers represent *SD*s. (b) The scatter plot of MA concerning the ACC to some special regions, including R AG, R SFG, L MFG, and R MFG. A single asterisk indicated *p* < .01 and double asterisks indicated *p* < .005. Bars indicate mean values, and whiskers represent *SEM*s. (c) The difference of MA in the anterior default mode subnetwork between responders and nonresponders. Key nodes are shown in red color. Green lines represent positive MA and blue lines represent negative MA. The larger the MA between them, the stronger the line. ACC, bilateral anterior cingulate cortex; L MFG, left middle frontal gyrus; MA, module allegiance; R AG, right angular gyrus; R MFG, right middle frontal gyrus; R SFG, right superior frontal gyrus

In further analyses, the interaction of these eight significantly discriminative regions was explored on a system or subsystem level to assess the differences between responders and nonresponders. The statistical *t* test showed that the MA of ACC with all other nodes was lower in patients with MDD comparing with healthy controls (*t* = 4.932, *df* = 67, *p* < .001, FDR‐corrected, 95% confidence interval [CI] [1.0327, 2.4368], Figure 3a), while responders possessed larger characteristic comparing with nonresponders (*t* = 3.045, *df* = 32, *p* = .005, FDR‐corrected, 95% CI [0.4525, 2.3101], Figure 3a). The statistical *t* test further suggested that MAs of ACC concerning other special nodes were significantly different between responders and nonresponders as well, including right angular gyrus (R AG, *t* = 2.410, *df* = 32, *p* = .022, uncorrected, 95% CI [0.0217, 0.2607], Figure 3b), right superior frontal gyrus (R SFG, *t* = 2.557, *df* = 32, *p* = .016, uncorrected, 95% CI [0.0388, 0.3460]), right angular gyrus (R AG, *t* = 2.356, *df* = 32, *p* = .025, uncorrected, 95% CI [0.0210, 0.2910]), left middle frontal gyrus (L MFG, *t* = 3.000, *df* = 32, *p* = .005, uncorrected, 95% CI [0.0808, 0.4235]), and RMFG (*t* = 2.421, *df* = 32, *p* = .022, uncorrected, 95% CI [0.0296, 0.3463]). Interestingly, these regions formed spatially distributed system whereby ACC was the structural core (Figure 3c). It suggests that ACC acts as a pivotal part in facilitating the communication with a network that is particularly germane to escitalopram treatment.

### 3.6 Multicenter comparison

**Figure 4:**
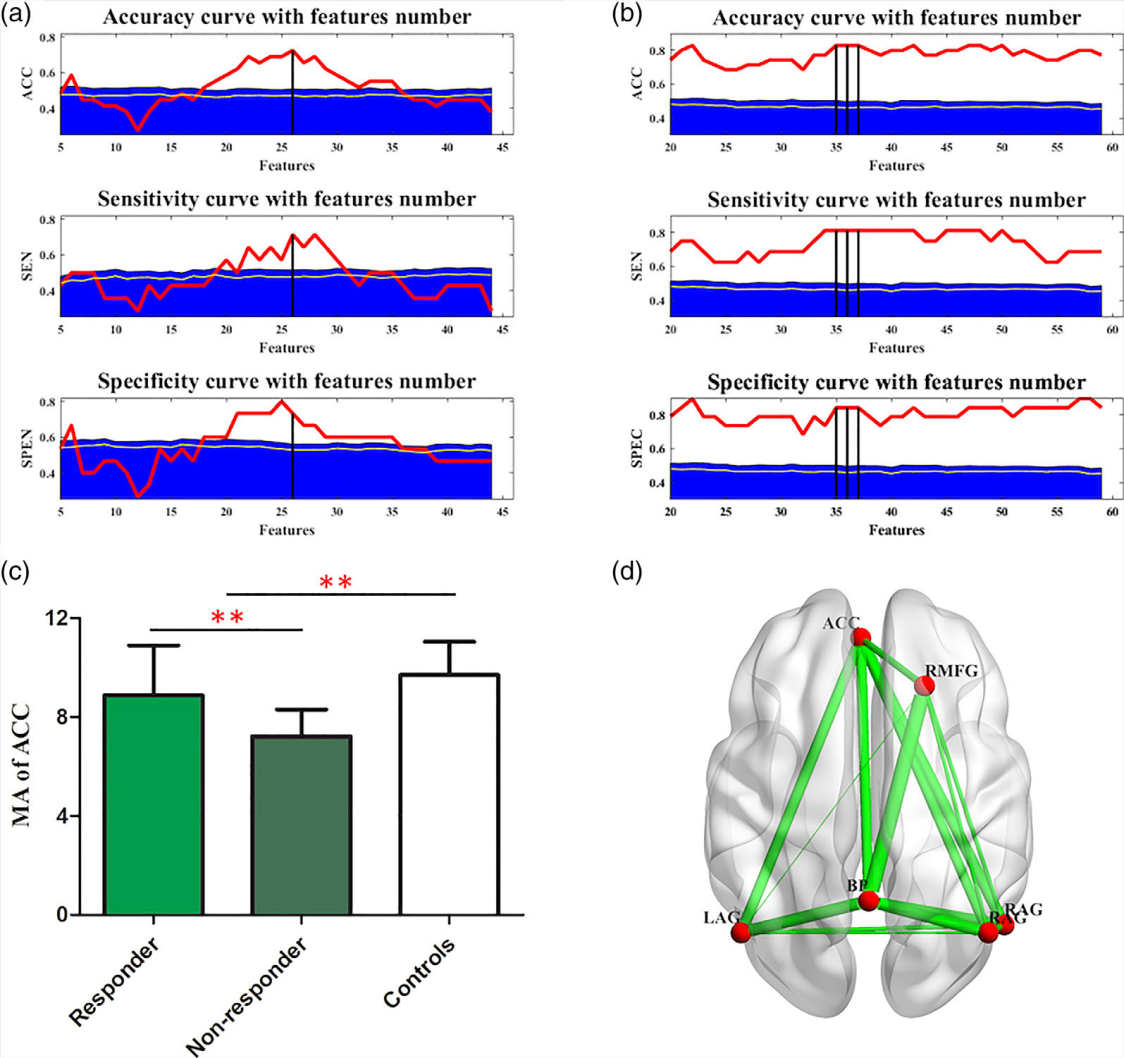
Multicenter comparison. The performances of SVM models in Peking University Institute of Mental Health (a) and Nanjing Drum Tower Hospital (b). The differences of MA matrices of subjects site 3 are showed in (c) and (d) (also see Supporting Information for site 2). Bars indicate mean values, and whiskers represent *SD*s. BP possessed another key hub properties which interacted closely with ACC (description similar to Figure 3). ACC, bilateral anterior cingulate cortex; BP, bilateral precuneus; L AG, left angular gyrus; MA, module allegiance; R AG, right angular gyrus; R MFG, right middle frontal gyrus; SVM, support vector machine

Parallel individual treatment response model for both Center 2 and Center 3 were trained and tested independently as well. The similar performances can be found in both centers as were showed in Figure 4. The real balanced accuracy was 82.86% in Drum Tower Hospital and 94.44% in Peking University Institute of Mental Health. The statistical *t* test showed that the MA of ACC (*t* = 3.418, *df* = 73, *p* = .001, 95% CI [0.5649, 2.1552], Figure 4c) was significantly different between patients and controls for all the subjects from these two sites. Moreover, statistical analyses showed that the MA of ACC (*t* = 3.143, *df* = 34, *p* = .004, 95% CI [0.5807, 2.7332], Figure 4c) was significantly different between responders and nonresponders mainly because of the interaction of ACC with several special nodes (see Figures S5 and S6 for each site, respectively). Besides, these regions formed a spatially distributed ACC‐hub DMN subnetwork (Figure 4d), implying that escitalopram has intervened efficiency via the ACC.

Besides above satisfying model performance in each site, we further tried the leave‐*one‐group*‐out analysis to test the site effects. We trained in two sites and tested in third one. The performance of leave‐*one‐group*‐out (69% for leaving Site 1 out, 71% for leaving Site 2 out, 72% for leaving Site 3 out) was comparable with that of the within‐group LOOCV performance (79%). It supported the robustness of our model frame across sites.

## 4 DISCUSSIONS

Using a baseline fMRI and SVM models, we presented the flexibility of some special regions, such as MFG, right insula, and ACC for accurate prediction of escitalopram treatment response. We specifically chose to characterize functional connection using MA since it displays behaviorally or pathophysiologically relevant interactions. A larger MA of ACC was found in responders compared to nonresponders. Further investigation revealed a system level dysfunction whereby the ACC was found to be the crucial discriminative region.

### 4.1 The flexible brain

A clear interaction between regions congruent with FC showed temporal changeability (Figure 1b,c). The flexible brain was also found in humans during the learning process (Bassett et al., 2011). Flexibility is deemed as a trustworthy indicator of a given subject's biological process in response to learning or neurorehabilitation. Treatment response of 8 weeks prescription with escitalopram could be predicted by the baseline flexibility of brain regions (Figure 2c,d), suggesting the flexible brain could be beneficial for depressed individuals' pharmacological intervention.

### 4.2 ACC effects on antidepressant outcome

The ACC is involved in the processing of specific modules which is responsible for reward anticipation (Bush et al., 2002), attention, error detection, conflict monitoring (Bush, Luu, & Posner, 2000; Shenhav, Botvinick, & Cohen, 2013), social cognition (Apps, Rushworth, & Chang, 2016; Tolomeo et al., 2016), and emotional response (Bush et al., 2000; Etkin, Büchel, & Gross, 2015; Tolomeo et al., 2016). Its abnormalities were associated with diverse symptoms such as suicidal thoughts (Holmes et al., 2017), negative bias, poor cognition, and comorbid anxiety in depression. Fox, Buckner, White, Greicius, and Pascualleone (2012) applied rTMS to depressed patients and found that a more anticorrelated FC of ACC predicted better treatment response. Weigand et al. (2017) also applied rTMS to predict antidepressant efficacy but targeted the subgenual ACC. In addition, CBT trials found that ACC hypoactivation was linked to better treatment outcomes (Ball, Stein, & Paulus, 2014). These findings confirmed that rTMS and CBT targeted directly or indirectly the ACC to rehabilitate the individuals who are predisposed to less activation (Fox et al., 2012).

In contrast, the most consistent finding in mediation studies was that greater ACC activation was associated with better outcomes (Ball et al., 2014). This finding indicated that medication treatment may not target the ACC activity and may therefore be most beneficial for individuals who exhibited robust pretreatment activation. Escitalopram, as a SSRIs, administration increases the level of 5‐hydroxytryptamine in the serotonergic system, including prefrontal cortical area, amygdala, ACC and hippocampus. Vai et al. (2016) found reduced effective connectivity from amygdala to the ventrolateral prefrontal cortex and to ACC, with an increased modulation of ACC to amygdala during fearful emotional stimuli in nonresponders to escitalopram monotherapy. Chakroborty et al. (2017) did not discover any significant differences in activation in the ACC after treatment. These findings suggested that frontal‐ACC circuit, not a local region, might provide a neuropathy mechanism that involved in recovery from depression.

Flexibility measures the attributes of a region that changes its modular allegiance which is motivated by top‐down connectivity (frontal‐ACC). Higher ACC modular allegiance was found in the responders than nonresponders, suggesting that top‐down influences might be biased by the pretreatment status of ACC which in turn can act as a predictive biomarker of effective response to SSRIs treatment.

### 4.3 The ACC and DMN

The DMN can be divided into the dorsal medial and medial temporal subsystem (Andrewshanna, Smallwood, & Spreng, 2014), which broadly overlaps with the anterior and posterior subsets (Buckner, Andrewshanna, & Schacter, 2008; Raichle et al., 2001). The part of ACC connecting with the medial prefrontal cortex is the main brain region of the anterior DMN subnetwork. In recent years, the dissociation of DMN is of great research interest in MDD. Li et al. (2013) found an increased FC within both anterior and posterior DMN, but only the posterior DMN was normalized after treatment. A study which investigated the relationships between DMN subsystems and rumination showed opposite connectivity alteration within different subnetworks (Zhu, Zhu, Shen, Liao, & Yuan, 2017). Consequently, DMN subsets may provide new insights into the pathophysiology and antidepressant response efficiency in MDD.

The different DMN subsystems, of which the ACC was a hub, distinctively impacted escitalopram treatment efficacy. As a result, the strong functional integration of ACC may be regarded as a predictor of escitalopram monotherapy outcome in major depressive patients. This implies that the pretreatment modular integration of ACC may be used to assess patients' recovery progress. Our findings have a huge potential to be replicated in the future.

### 4.4 Exploring the robust biomarks for treatment response prediction

Many studies demonstrated that even a single dose of escitalopram is sufficient to alter the brain's functional anatomy even in a short term (Komulainen et al., 2018). Studies of brain response to a single dose, such as escitalopram, would seem to be noteworthy and relevant. Barron et al. (2018) applied a cross‐validated predictive model to classify pharmacologic effect across 11 task‐based fMRI data sets (306 samples). However, they failed to classify if antidepressants modulate brain responses to the emotional faces task and achieved only limited evidence in a consistent manner. It may partly be explained by different drug administrations and patient populations across datasets. Furthermore, more standardized implementation of the task protocol is in demand in the future work. In this consideration, measuring the changes in emotional processing may weak the individual effect on the task protocol and then provide a sensitive early measure of antidepressant efficacy for individual patients (Browning et al., 2019).

Our consistent performance over data sets suggested the potential ability of resting state fMRI over exploring the clinical consistent biomarks. The results in relation to ACC‐core subnetwork were repeated with data from Drum Tower Hospital (Samples 2) and Peking University Institute of Mental Health (Samples 3). Predicting of the treatment response performs well within‐data set Leave‐One‐Sample‐Out validation and cross‐data set Leave‐One‐Group‐Out validation. The significant difference of overall MA in ACC did not reoccur in Sample 2. It is possibly derived from heterogeneity of the subjects, whereby the averaged MA of ACC concerning other regions cannot be sensitive enough to detect the individual treatment response. However, each MA value between ACC to some special regions was still significant. Therefore, we can conclude the important role of ACC‐core subnetwork in treatment response prediction.

The main limitation of this experiment is a relatively small sample size. Our results should be further validated by larger cohorts together with multimodal data and other therapy methods. One limitation of this study in the design was an open‐labeled and not placebo‐controlled. The response to antidepressant treatment could be interpreted by a combination of specific and nonspecific effects, which in addition to placebo neurobiological effects, may include variations in the natural history of illness, regression to the mean, and reporting biases.

In conclusion, the present study is the first to demonstrate individual response of MDD patients with escitalopram by use of baseline resting state fMRI and then validate over different datasets. A more advanced analysis showed significant hyperinteraction within the ACC‐core subnetwork in first‐episode, drug‐naïve responders. We confirm that ACC could serve as a predictor for better treatment outcomes in individuals prescribed with escitalopram monotherapy. Overall, our findings suggest that there is a huge potential in combining fMRI data with machine learning techniques. This technique will further evolve to identify biomarkers which may be used as a reliable biomarker for prognosis, optimizing therapeutic decisions and assisting psychiatric research of depression.

## CONFLICT OF INTEREST

The authors have no conflict of interest to declare.

## Supporting information


**Appendix S1.** Supporting Information.

## Data Availability

Data and codes used for the present analyses are available from the corresponding author

## References

[hbm24872-bib-0001] Allen, E. A. , Damaraju, E. , Plis, S. M. , Erhardt, E. B. , Eichele, T. , & Calhoun, V. D. (2014). Tracking whole‐brain connectivity dynamics in the resting state. Cerebral Cortex, 24(3), 663–676. 23146964 10.1093/cercor/bhs352 PMC3920766

[hbm24872-bib-0002] Andrewshanna, J. R. , Smallwood, J. , & Spreng, R. N. (2014). The default network and self‐generated thought: Component processes, dynamic control, and clinical relevance. Annals of the New York Academy of Sciences, 1316(1), 29. 24502540 10.1111/nyas.12360 PMC4039623

[hbm24872-bib-0003] Apps, M. A. J. , Rushworth, M. F. S. , & Chang, S. W. C. (2016). The anterior cingulate gyrus and social cognition: Tracking the motivation of others. Neuron, 90(4), 692–707. 27196973 10.1016/j.neuron.2016.04.018 PMC4885021

[hbm24872-bib-0004] Ball, T. M. , Stein, M. B. , & Paulus, M. P. (2014). Toward the application of functional neuroimaging to individualized treatment for anxiety and depression. Depression and Anxiety, 31(11), 920–933. 25407582 10.1002/da.22299

[hbm24872-bib-0005] Barron, D. S. , Salehi, M. , Browning, M. , Harmer, C. J. , Constable, R. T. , & Duff, E. (2018). Exploring the prediction of emotional valence and pharmacologic effect across fMRI studies of antidepressants. Neuroimage: Clinical, 20, 407–414. 10.1016/j.nicl.2018.08.016 30128279 PMC6096053

[hbm24872-bib-0006] Bassett, D. S. , Wymbs, N. F. , Porter, M. A. , Mucha, P. J. , Carlson, J. M. , & Grafton, S. T. (2011). Dynamic reconfiguration of human brain networks during learning. Proceedings of the National Academy of Sciences of the United States of America, 108(18), 7641–7646. 21502525 10.1073/pnas.1018985108 PMC3088578

[hbm24872-bib-0007] Bassett, D. S. , Yang, M. , Wymbs, N. F. , & Grafton, S. T. (2015). Learning‐induced autonomy of sensorimotor systems. Nature Neuroscience, 18(5), 744–751. 25849989 10.1038/nn.3993 PMC6368853

[hbm24872-bib-0059] Braun, U. , Schäfer, A. , Walter, H. , Erk, S. , Romanczuk‐Seiferth, N. , Haddad, L. , … Bassett, D. S. (2015). Dynamic reconfiguration of frontal brain networks during executive cognition in humans. Proceedings of the National Academy of Sciences of the United States of America, 112(37), 11678–11683. 10.1073/pnas.1422487112 26324898 PMC4577153

[hbm24872-bib-0060] Braun, U. , Schäfer, A. , Bassett, D. S. , Rausch, F. , Schweiger, J. I. , Bilek, E. , … Tost, H. (2016). Dynamic brain network reconfiguration as a potential schizophrenia genetic risk mechanism modulated by NMDA receptor function. Proceedings of the National Academy of Sciences of the United States of America, 113(44), 12568–12573. 10.1073/pnas.1608819113 27791105 PMC5098640

[hbm24872-bib-0009] Browning, M. , Kingslake, J. , Dourish, C. T. , Goodwin, G. M. , Harmer, C. J. , & Dawson, G. R. (2019). Predicting treatment response to antidepressant medication using early changes in emotional processing. European Neuropsychopharmacology, 29(1), 66–75. 10.1016/j.euroneuro.2018.11.1102 30473402

[hbm24872-bib-0010] Buckner, R. L. , Andrewshanna, J. R. , & Schacter, D. L. (2008). The brain's default network: Anatomy, function, and relevance to disease. Annals of the New York Academy of Sciences, 1124(1), 1. 18400922 10.1196/annals.1440.011

[hbm24872-bib-0011] Bush, G. , Luu, P. , & Posner, M. I. (2000). Cognitive and emotional influences in anterior cingulate cortex. Trends in Cognitive Sciences, 4(6), 215. 10827444 10.1016/s1364-6613(00)01483-2

[hbm24872-bib-0012] Bush, G. , Vogt, B. A. , Holmes, J. , Dale, A. M. , Greve, D. , Jenike, M. A. , & Rosen, B. R. (2002). Dorsal anterior cingulate cortex: A role in reward‐based decision making. Proceedings of the National Academy of Sciences of the United States of America, 99(1), 523–528. 11756669 10.1073/pnas.012470999 PMC117593

[hbm24872-bib-0061] Cawley, G. C. , & Talbot, N. L. C. (2004). Fast exact leave‐one‐out cross‐validation of sparse least‐squares support vector machines. Neural Networks, 17(10), 1467–1475. 10.1016/j.neunet.2004.07.002 15541948

[hbm24872-bib-0062] Chakroborty, S. , Geisbush, T. R. , Dale, E. , Pehrson, A. L. , Sánchez, C. , & West, A. R. (2017). Impact of Vortioxetine on Synaptic Integration in Prefrontal‐Subcortical Circuits: Comparisons with Escitalopram. Frontiers in Pharmacology, 8, 764–764. 10.3389/fphar.2017.00764 29123483 PMC5662919

[hbm24872-bib-0013] Cipriani, A. , Furukawa, T. A. , Salanti, G. , Geddes, J. R. , Higgins, J. P. , Churchill, R. , … Mcguire, H. (2009). Comparative efficacy and acceptability of 12 new‐generation antidepressants: A multiple‐treatments meta‐analysis. Lancet, 373(9665), 746–758. 19185342 10.1016/S0140-6736(09)60046-5

[hbm24872-bib-0063] Ciric, R. , Wolf, D. H. , Power, J. D. , Roalf, D. R. , Baum, G. L. , Ruparel, K. , … Satterthwaite, T. D. (2017). Benchmarking of participant‐level confound regression strategies for the control of motion artifact in studies of functional connectivity. Neuroimage, 154, 174–187. 10.1016/j.neuroimage.2017.03.020 28302591 PMC5483393

[hbm24872-bib-0014] Crane, N. A. , Jenkins, L. M. , Bhaumik, R. , Dion, C. , Gowins, J. R. , Mickey, B. J. , … Langenecker, S. A. (2017). Multidimensional prediction of treatment response to antidepressants with cognitive control and functional MRI. Brain, 140(Pt. 2), 472–486. 28122876 10.1093/brain/aww326 PMC5278310

[hbm24872-bib-0015] Dichter, G. S. , Gibbs, D. , & Smoski, M. J. (2015). A systematic review of relations between resting‐state functional‐MRI and treatment response in major depressive disorder. Journal of Affective Disorders, 172, 8–17. 25451389 10.1016/j.jad.2014.09.028 PMC4375066

[hbm24872-bib-0064] Drysdale, A. T. , Grosenick, L. , Downar, J. , Dunlop, K. , Mansouri, F. , Meng, Y. , … Liston, C. (2017). Erratum: Resting‐state connectivity biomarkers define neurophysiological subtypes of depression. Nature Medicine, 23(2), 264–264. 10.1038/nm0217-264d PMC5639473 28170383

[hbm24872-bib-0016] Dunlop, B. W. , Rajendra, J. K. , Craighead, W. E. , Kelley, M. E. , Mcgrath, C. L. , Choi, K. S. , … Mayberg, H. S. (2017). Functional connectivity of the subcallosal cingulate cortex and differential outcomes to treatment with cognitive‐behavioral therapy or antidepressant medication for major depressive disorder. American Journal of Psychiatry, 174(6), 533–545. 28335622 10.1176/appi.ajp.2016.16050518 PMC5453828

[hbm24872-bib-0017] Etkin, A. , Büchel, C. , & Gross, J. J. (2015). The neural bases of emotion regulation. Nature Reviews Neuroscience, 16(11), 693–700. 26481098 10.1038/nrn4044

[hbm24872-bib-0018] Fox, M. D. , Buckner, R. L. , White, M. P. , Greicius, M. D. , & Pascualleone, A. (2012). Efficacy of transcranial magnetic stimulation targets for depression is related to intrinsic functional connectivity with the subgenual cingulate. Biological Psychiatry, 72(7), 595–603. 22658708 10.1016/j.biopsych.2012.04.028 PMC4120275

[hbm24872-bib-0019] Global Burden of Disease Study . (2015). Global, regional, and national incidence, prevalence, and years lived with disability for 301 acute and chronic diseases and injuries in 188 countries, 1990–2013: A systematic analysis for the Global Burden of Disease Study 2013. The Lancet, 386(9995), 743–800. 10.1016/S0140-6736(15)60692-4 PMC4561509 26063472

[hbm24872-bib-0020] Godlewska, B. R. , Browning, M. , Norbury, R. , Igoumenou, A. , Cowen, P. J. , & Harmer, C. J. (2018). Predicting treatment response in depression: The role of anterior cingulate cortex. International Journal of Neuropsychopharmacology, 21(11), 988–996. 10.1093/ijnp/pyy069 30124867 PMC6209854

[hbm24872-bib-0021] Guo, W. , Liu, F. , Xue, Z. , Gao, K. , Liu, Z. , Xiao, C. , … Zhao, J. (2013). Abnormal resting‐state cerebellar‐cerebral functional connectivity in treatment‐resistant depression and treatment sensitive depression. Progress in Neuro‐Psychopharmacology and Biological Psychiatry, 44(5), 51–57. 23352887 10.1016/j.pnpbp.2013.01.010

[hbm24872-bib-0022] Hamilton, M. (1967). Development of a rating scale for primary depressive illness. British Journal of Social and Clinical Psychology, 6(4), 278–296. 6080235 10.1111/j.2044-8260.1967.tb00530.x

[hbm24872-bib-0023] Hermundstad, A. M. , Brown, K. S. , Bassett, D. S. , Aminoff, E. M. , Frithsen, A. , Johnson, A. , … Carlson, J. M. (2014). Structurally‐constrained relationships between cognitive states in the human brain. PLoS Computational Biology, 10(5), e1003591 10.1371/journal.pcbi.1003591 24830758 PMC4022461

[hbm24872-bib-0024] Holmes, S. E. , Hinz, R. , Conen, S. , Gregory, C. J. , Matthews, J. C. , Anton‐Rodriguez, J. M. , … Talbot, P. S. (2017). Elevated translocator protein in anterior cingulate in major depression and a role for inflammation in suicidal thinking: A positron emission tomography study. Biological Psychiatry. 83(1), 61–69. 10.1016/j.biopsych.2017.08.005 28939116

[hbm24872-bib-0025] Holtzheimer, P. E. , & Mayberg, H. S. (2011). Stuck in a rut: Rethinking depression and its treatment. Trends in Neurosciences, 34(1), 1–9. 21067824 10.1016/j.tins.2010.10.004 PMC3014414

[hbm24872-bib-0026] Katikireddi, S. V. (2017). Global, regional, and national incidence and prevalence, and years lived with disability for 328 diseases and injuries in 195 countries, 1990–2016: A systematic analysis for the Global Burden of Disease Study 2016. 390(10100), 1211–1259. 10.1016/S0140-6736(17)32154-2 PMC5605509 28919117

[hbm24872-bib-0027] Komulainen, E. , Heikkila, R. , Nummenmaa, L. , Raij, T. T. , Harmer, C. J. , Isometsa, E. , & Ekelund, J. (2018). Short‐term escitalopram treatment normalizes aberrant self‐referential processing in major depressive disorder. Journal of Affective Disorders, 236, 222–229. 10.1016/j.jad.2018.04.096 29747140

[hbm24872-bib-0028] Kozel, F. A. , Rao, U. , Lu, H. , Nakonezny, P. A. , Grannemann, B. , McGregor, T. , … Trivedi, M. H. (2011). Functional connectivity of brain structures correlates with treatment outcome in major depressive disorder. Frontiers in Psychiatry, 2, 7. 21556277 10.3389/fpsyt.2011.00007 PMC3089997

[hbm24872-bib-0029] Li, B. , Liu, L. , Friston, K. J. , Shen, H. , Wang, L. , Zeng, L. L. , & Hu, D. (2013). A treatment‐resistant default mode subnetwork in major depression. Biological Psychiatry, 74(1), 48–54. 23273724 10.1016/j.biopsych.2012.11.007

[hbm24872-bib-0030] Listed, N. (2000). Practice guideline for the treatment of patients with major depressive disorder (revision). American Psychiatric Association. American Journal of Psychiatry, 157(4 Suppl.), 1. 10767867

[hbm24872-bib-0031] Ma, C. , Ding, J. , Li, J. , Guo, W. , Long, Z. , Liu, F. , … Chen, H. (2012). Resting‐state functional connectivity bias of middle temporal gyrus and caudate with altered gray matter volume in major depression. PLoS One, 7(9), e45263. 23028892 10.1371/journal.pone.0045263 PMC3454420

[hbm24872-bib-0032] Maier, W. , Buller, R. , Philipp, M. , & Heuser, I. (1988). The Hamilton anxiety scale: Reliability, validity and sensitivity to change in anxiety and depressive disorders. Journal of Affective Disorders, 14(1), 61–68. 2963053 10.1016/0165-0327(88)90072-9

[hbm24872-bib-0033] Mohr, H. , Wolfensteller, U. , & Ruge, H. (2017). Large‐scale coupling dynamics of instructed reversal learning. NeuroImage, 167, 237–246. 10.1016/j.neuroimage.2017.11.049. 29175610

[hbm24872-bib-0034] Mucha, P. J. , & Onnela, J. P. (2010). Community structure in time‐dependent, multiscale, and multiplex networks. Science, 328(5980), 876–878. 20466926 10.1126/science.1184819

[hbm24872-bib-0035] Papakostas, G. I. , Thase, M. E. , Fava, M. , Nelson, J. C. , & Shelton, R. C. (2007). Are antidepressant drugs that combine serotonergic and noradrenergic mechanisms of action more effective than the selective serotonin reuptake inhibitors in treating major depressive disorder? A meta‐analysis of studies of newer agents. Biological Psychiatry, 62(11), 1217–1227. 17588546 10.1016/j.biopsych.2007.03.027

[hbm24872-bib-0036] Peng, H. , Long, F. , & Ding, C. (2005). Feature selection based on mutual information: Criteria of max‐dependency, max‐relevance, and min‐redundancy. IEEE Transactions on Pattern Analysis & Machine Intelligence, 27(8), 1226–1238. 16119262 10.1109/TPAMI.2005.159

[hbm24872-bib-0037] Phillips, M. L. , Chase, H. W. , Sheline, Y. I. , Etkin, A. , Almeida, J. R. , Deckersbach, T. , & Trivedi, M. H. (2015). Identifying predictors, moderators, and mediators of antidepressant response in major depressive disorder: Neuroimaging approaches. American Journal of Psychiatry, 172(2), 124–138. 25640931 10.1176/appi.ajp.2014.14010076 PMC4464814

[hbm24872-bib-0038] Raichle, M. E. , Macleod, A. M. , Snyder, A. Z. , Powers, W. J. , Gusnard, D. A. , & Shulman, G. L. (2001). A default mode of brain function. Proceedings of the National Academy of Sciences of the United States of America, 98(2), 676–682. 11209064 10.1073/pnas.98.2.676 PMC14647

[hbm24872-bib-0039] Sanchez, C. , Reines, E. H. , & Montgomery, S. A. (2014). A comparative review of escitalopram, paroxetine, and sertraline: Are they all alike? International Clinical Psychopharmacology, 29(4), 185–196. 24424469 10.1097/YIC.0000000000000023 PMC4047306

[hbm24872-bib-0040] Scheinost, D. , Noble, S. , Horien, C. , Greene, A. S. , Lake, E. M. R. , Salehi, M. , … Constable, R. T. (2019). Ten simple rules for predictive modeling of individual differences in neuroimaging. NeuroImage, 193, 35–45. 10.1016/j.neuroimage.2019.02.057 30831310 PMC6521850

[hbm24872-bib-0041] Schrouff, J. , Rosa, M. J. , Rondina, J. M. , Marquand, A. F. , Chu, C. , Ashburner, J. , … Mourão‐Miranda, J. (2013). PRoNTo: Pattern recognition for neuroimaging toolbox. Neuroinformatics, 11(3), 319–337. 23417655 10.1007/s12021-013-9178-1 PMC3722452

[hbm24872-bib-0042] Shao, J. , Dai, Z. , Zhu, R. , Wang, X. , Tao, S. , Bi, K. , … Lu, Q. (2019). Early identification of bipolar from unipolar depression before manic episode: Evidence from dynamic rfMRI. Bipolar Disorders. 10.1111/bdi.12819 31407477

[hbm24872-bib-0043] Sheehan, D. V. , Lecrubier, Y. , Sheehan, K. H. , Amorim, P. , Janavs, J. , Weiller, E. , … Dunbar, G. C. (1998). The MINI international neuropsychiatric interview (MINI): The development and validation of a structured interview for DSM‐IV and ICD‐10. Journal of Clinical Psychiatry, 59(Suppl. 20), 22. 9881538

[hbm24872-bib-0044] Shenhav, A. , Botvinick, M. M. , & Cohen, J. D. (2013). The expected value of control: An integrative theory of anterior cingulate cortex function. Neuron, 79(2), 217–240. 23889930 10.1016/j.neuron.2013.07.007 PMC3767969

[hbm24872-bib-0045] Storey, J. D. (2003). The positive false discovery rate: A Bayesian interpretation and the q‐value. Annals of Statistics, 31(6), 2013–2035.

[hbm24872-bib-0046] Tao, H. , Guo, S. , Ge, T. , Kendrick, K. M. , Xue, Z. , Liu, Z. , & Feng, J. (2013). Depression uncouples brain hate circuit. Molecular Psychiatry, 18(1), 101–111. 10.1038/mp.2011.127 21968929 PMC3526729

[hbm24872-bib-0047] Tian, S. , Chattun, M. R. , Zhang, S. , Bi, K. , Tang, H. , Yan, R. , … Lu, Q. (2019). Dynamic community structure in major depressive disorder: A resting‐state MEG study. Progress in Neuro‐Psychopharmacology and Biological Psychiatry, 92, 39–47. 10.1016/j.pnpbp.2018.12.006 30572002

[hbm24872-bib-0048] Tolomeo, S. , Christmas, D. , Jentzsch, I. , Johnston, B. , Sprengelmeyer, R. , Matthews, K. , & Steele, J. D. (2016). A causal role for the anterior mid‐cingulate cortex in negative affect and cognitive control. Brain, 139(Pt. 6), 1844–1854. 27190027 10.1093/brain/aww069

[hbm24872-bib-0049] Trivedi, M. H. , Rush, A. J. , Wisniewski, S. R. , Nierenberg, A. A. , Warden, D. , Ritz, L. , … McGrath, P. J. (2005). Evaluation of outcomes with citalopram for depression using measurement‐based care in STAR*D: Implications for clinical practice. American Journal of Psychiatry, 163(1), 28. 10.1176/appi.ajp.163.1.28 16390886

[hbm24872-bib-0065] Vai, B. , Bulgarelli, C. , Godlewska, B. R. , Cowen, P. J. , Benedetti, F. , & Harmer, C. J. (2016). Fronto‐limbic effective connectivity as possible predictor of antidepressant response to SSRI administration. European Neuropsychopharmacology, 26(12), 2000–2010. 10.1016/j.euroneuro.2016.09.640 27756525

[hbm24872-bib-0050] Varoquaux, G. , Raamana, P. R. , Engemann, D. A. , Hoyos‐Idrobo, A. , Schwartz, Y. , & Thirion, B. (2016). Assessing and tuning brain decoders: Cross‐validation, caveats, and guidelines. NeuroImage, 145, 166–179. 10.1016/j.neuroimage.2016.10.038. 27989847

[hbm24872-bib-0051] Wang, H. R. , Woo, Y. S. , Ahn, H. S. , Ahn, I. M. , Kim, H. J. , & Bahk, W. M. (2015). Can atypical antipsychotic augmentation reduce subsequent treatment failure more effectively among depressed patients with a higher degree of treatment resistance? A meta‐analysis of randomized controlled trials. International Journal of Neuropsychopharmacology, 18(8), pyv023 10.1093/ijnp/pyv023. 25770098 PMC4571632

[hbm24872-bib-0052] Wang, L.‐J. , Kuang, W.‐H. , Xu, J.‐J. , Lei, D. , & Yang, Y.‐C. (2014). Resting‐state brain activation correlates with short‐time antidepressant treatment outcome in drug‐naive patients with major depressive disorder. Journal of International Medical Research, 42(4), 966–975. 24898399 10.1177/0300060514533524

[hbm24872-bib-0053] Weigand, A. , Horn, A. , Caballero, R. , Cooke, D. , Stern, A. P. , Taylor, S. F. , … Fox, M. D. (2017). Prospective validation that subgenual connectivity predicts antidepressant efficacy of transcranial magnetic stimulation sites. Biological Psychiatry, 84(1), 28–37. 29274805 10.1016/j.biopsych.2017.10.028 PMC6091227

[hbm24872-bib-0054] Whooley, M. A. , & Simon, G. E. (2000). Managing depression in medical outpatients. New England Journal of Medicine, 343(26), 1942–1950. 11136266 10.1056/NEJM200012283432607

[hbm24872-bib-0055] Williams, L. M. (2017). Getting personalized: Brain scan biomarkers for guiding depression interventions. American Journal of Psychiatry, 174(6), 503–505. 28565957 10.1176/appi.ajp.2017.17030314

[hbm24872-bib-0056] Yan, C. , & Zang, Y. (2010). DPARSF: A MATLAB toolbox for "pipeline" data analysis of resting‐state fMRI. Frontiers in Systems Neuroscience, 4(13), 13. 20577591 10.3389/fnsys.2010.00013 PMC2889691

[hbm24872-bib-0057] Zheng, H. , Li, F. , Bo, Q. , Li, X. , Yao, L. , Yao, Z. , … Wu, X. (2017). The dynamic characteristics of the anterior cingulate cortex in resting‐state fMRI of patients with depression. Journal of Affective Disorders, 227, 391–397. 29154155 10.1016/j.jad.2017.11.026

[hbm24872-bib-0058] Zhu, X. , Zhu, Q. , Shen, H. , Liao, W. , & Yuan, F. (2017). Rumination and default mode network subsystems connectivity in first‐episode, drug‐naive young patients with major depressive disorder. Scientific Reports, 7, 43105. 28225084 10.1038/srep43105 PMC5320523

