# Supplementary material for: Predicting escitalopram monotherapy response in depression: The role of anterior cingulate cortex
Source: Hum Brain Mapp. 2019 Nov 22;41(5):1249–60. doi: 10.1002/hbm.24872 (PMC7268019; doi:10.1002/hbm.24872)
Supplement: Supplementary file 1 — Appendix S1. Supporting Information. [file HBM-41-1249-s001.docx]

**Supplementary information for**

**Pretreatment modularity of anterior cingulate cortex predicts escitalopram monotherapy response in depression**

**Methods 1; tables 1; Figures 6**

**Methods**

**FC estimation and temporal variability**

We determined the whole brain dynamic FC by employing Pearson correlation on a previously published brain template comprising of 95 regions of interest (ROI) in six brain networks (Allen, et al., 2014). The width of rectangular window was 45TRs, with a 1TR step size. Following the graphical LASSO methods (Friedman, et al., 2007), a penalty of L1 was applied to promote sparsity and stability of dynamic FC within short time window segments. For each subject, this estimation yielded 99 (95×95 ROIs) weighted matrices tracing FC in each time window.

**Traits of Community Detection**

The resulting correlation matrices of each subjects were partitioned into time-dependent communities using a multilayer community detection algorithm which was based on a Louvain-like Locally greedy optimization method in the view of maximizing the modality quality function(Mucha and Onnela, 2010). As a result, the 99 weighted adjacency matrices were combined into a rank 3 adjacency tensor for representing time-dependent or multilayer networks. One can thereby obtain a multilayer modularity:

**$Q=\frac{1}{2\mu}\sum_{ijlr} \{(A_{ijl}-\gamma_{l}P_{ijl})\delta_{ij}+\delta_{ij}\omega_{ijr}\}\delta(g_{il},g_{jr})$** (1)

where *A_ijl_* is the adjacency matrix of layer *l* and the element *P_ijl_* represents the optimization components according to Newman-Girvan null model; *γ_l_* is the structural resolution parameter of layer *l*, *ω_jlr_* is the connection strength, we let *ω_jlr_* ≡ *ω* = 0.25 for neighboring layers and *γ_l_* ≡ *γ* = 1, *δ (g_il,_ g_jr_)* = 1 if *g_il_ = g_jr_* and it equals 0 otherwise; the total edge weight on the system is.

 (2)

$K_{il}^{\pm} =\sum_{j} A_{ijl}^{\pm}$ (3)

$m_{l}^{\pm} =\sum_{ij} A_{ijl}^{\pm}$ (4)

‘+’ and ‘−’ superscripts denote all positive and negative connections, respectively.

This procedure provided the communities’ configurations for each region and each time window, thereby designating the module allegiance. Owing to the greedy heuristic algorithm and the near-degeneracy of the maximization the quality function, each independent run produced a slightly different configuration of putative functional modules. Therefore, we repeated the modularity optimization algorithm 100 times for each subject.

**Reference**

Allen, E.A., Damaraju, E., Plis, S.M., Erhardt, E.B., Eichele, T., Calhoun, V.D. (2014) Tracking Whole-Brain Connectivity Dynamics in the Resting State. Cereb. Cortex, 24:663.

Friedman, J., Hastie, T., Tibshirani, R. (2007) Sparse inverse covariance estimation with the graphical lasso. Biostatistics, 9:432.

Mucha, P.J., Onnela, J.P. (2010) Community structure in time-dependent, multiscale, and multiplex networks. Science, 328:876-8.

**Tables and Figures**

Table S1 the demographic characteristics for all participants

|  | Patients with MDD | Healthy controls | *p* |
| --- | --- | --- | --- |
| Numbers of subjects | 34/36/36 | 35/35/39 | —— |
| Age  (years) | 33.82.74±11.10  /30.56±7.22  /33.88±14.34 | 35.11±11.31  /33.89±10.59  /34.30±10.67 | 0.849/0.642/0.846^a^ |
| Education (years) | 12.41±2.33  /14.153±3.72  /13.31±2.81 | 13.48±2.11  /14.69±2.28  /14.44±2.42 | 0.066/0.166/0.108^a^ |
| Gender  (male /female) | 20M14F/18M18F/17M19F | 15M22F/16M19F/18M21F | 0.124/0.718/0.926^b^ |
| Handedness  (left or right) | 0L34R/0L36R/0L36R | 0L35R/0L35R/0L39R | >0.999^b^ |

Values shown are listed as Nanjing Brain Hospital/ Nanjing Drum Tower Hospital/ Peking University Institute of Mental Health (mean±SD).

a: Two-sample t-test; b: Pearson Chi-square test

Supplementary Table S2 Definition of intrinsic networks by node location and MNI coordinates

| **Regional Name** | **MNI** | | |
| --- | --- | --- | --- |
|  | **X** | **Y** | **Z** |
| Subcortical networks | | | |
| R_caudate_nucleus | 12 | 0 | 15 |
| L_caudate_nucleus | -9 | 0 | 15 |
| R_putamen | 21 | 12 | -6 |
| L putamen | -21 | 9 | -3 |
| R putamen | 27 | 6 | -3 |
| L putamen | -30 | -3 | 0 |
| R thalamus | 9 | -15 | 6 |
| L thalamus | -9 | -18 | 6 |
| Auditory networks | | | |
| L superior temporal gyrus | -51 | -33 | 18 |
| R superior temporal gyrus | 51 | -30 | 21 |
| R superior temporal gyrus | 63 | -24 | 0 |
| L superior temporal gyrus | -57 | -21 | 3 |
| Somatomotor networks | | | |
| R postcentral gyrus | 57 | -6 | 27 |
| L postcentral gyrus | -54 | -9 | 30 |
| R cerebellum (VI) | 18 | -66 | -18 |
| L cerebellum (VI) | -18 | -63 | -21 |
| R postcentral gyrus | 42 | -24 | 60 |
| L cerebellum (VI) | -24 | -51 | -24 |
| L postcentral gyrus | -42 | -24 | 57 |
| R cerebellum (VI) | 21 | -51 | -24 |
| L supplementary motor area | -3 | -12 | 54 |
| Bi supplementary motor area | 3 | -3 | 48 |
| Bi paracentral lobule | -3 | -24 | 66 |
| L postcentral gyrus | -57 | -24 | 36 |
| R postcentral gyrus | 57 | -21 | 39 |
| Bi paracentral lobule | -18 | -9 | 66 |
| R superior parietal lobule | 18 | -54 | 66 |
| L superior parietal lobule | -21 | -45 | 63 |
| Visual networks | | | |
| L middle temporal gyrus | -48 | -72 | 9 |
| R middle temporal gyrus | 48 | -63 | 9 |
| R lingual gyrus | 21 | -72 | -6 |
| L lingual gyrus | -18 | -75 | -6 |
| Bi cuneus | 0 | -84 | 24 |
| L calcarine gyrus | -9 | -69 | 9 |
| R calcarine gyrus | 15 | -66 | 9 |
| Bi cuneus | 3 | -87 | 3 |
| L fusiform gyrus | -27 | -48 | -12 |
| R fusiform gyrus | 30 | -42 | -15 |
| R middle occipital gyrus | 36 | -84 | 6 |
| L middle occipital gyrus | -27 | -90 | 0 |
| R middle occipital gyrus | 30 | -93 | 0 |
| L middle occipital gyrus | -27 | -96 | -3 |
| L superior occipital gyrus | -36 | -81 | 30 |
| R superior occipital gyrus | 39 | -75 | 36 |
| Cognitive control networks | | | |
| R inferior parietal lobule | 45 | -39 | 51 |
| R middle frontal gyrus | 48 | 42 | 15 |
| R inferior frontal gyrus | 54 | 9 | 24 |
| R inferior temporal gyrus | 57 | -54 | -9 |
| L inferior temporal gyrus | -51 | -63 | -9 |
| R inferior temporal gyrus | 51 | -60 | -9 |
| L precentral gyrus | -48 | -6 | 48 |
| R precentral gyrus | 51 | -6 | 48 |
| L supplementary motor area | 0 | 0 | 66 |
| Bi middle cingulate cortex | 3 | 18 | 39 |
| R insula | 45 | -3 | 3 |
| L insula | -45 | -6 | 3 |
| L inferior parietal lobule | -42 | -51 | 51 |
| L middle frontal gyrus | -48 | 33 | 21 |
| L inferior temporal gyrus | -57 | -54 | -12 |
| R middle frontal gyrus | 45 | 15 | 33 |
| L middle frontal gyrus | -42 | 9 | 33 |
| R inferior frontal gyrus | 48 | 39 | 0 |
| L inferior frontal gyrus | -48 | 33 | 9 |
| R insula | 33 | 24 | -6 |
| L insula | -30 | 24 | -3 |
| R medial frontal gyrus | 6 | 30 | 39 |
| R inferior parietal lobule | 57 | -45 | 42 |
| L inferior parietal lobule | -57 | -45 | 39 |
| R middle frontal gyrus | 30 | 54 | 6 |
| L middle frontal gyrus | -30 | 54 | 12 |
| Bi supplementary motor area | -3 | 15 | 60 |
| R superior temporal gyrus | 57 | -48 | 15 |
| R inferior frontal gyrus | 51 | 30 | -3 |
| R ParaHippocampal gyrus | 24 | -18 | -21 |
| L ParaHippocampal gyrus | -21 | -18 | -21 |
| Default−mode networks | | | |
| Bi precuneus | -6 | -72 | 39 |
| Bi middle cingulate cortex | 0 | -24 | 30 |
| Bi precuneus | 0 | -54 | 51 |
| L posterior cingulate | -12 | -57 | 15 |
| R posterior cingulate | 15 | -54 | 15 |
| Bi posterior cingulate cortex | 3 | -36 | 27 |
| R angular gyrus | 45 | -66 | 42 |
| R Precuneus | 6 | -63 | 39 |
| R superior frontal gyrus | 24 | 33 | 54 |
| Bi anterior cingulate cortex | -3 | 45 | 3 |
| L angular gyrus | -48 | -66 | 33 |
| L Precuneus | 0 | -60 | 33 |
| R angular gyrus | 51 | -63 | 30 |
| L superior frontal gyrus | -18 | 39 | 51 |
| L medial frontal gyrus | -3 | 51 | -6 |
| L middle frontal gyrus | -24 | 24 | 48 |
| L superior frontal gyrus | 0 | 33 | 48 |
| R middle frontal gyrus | 21 | 27 | 45 |
| L middle temporal gyrus | -57 | -39 | -3 |
| L inferior frontal gyrus | -54 | 18 | 12 |


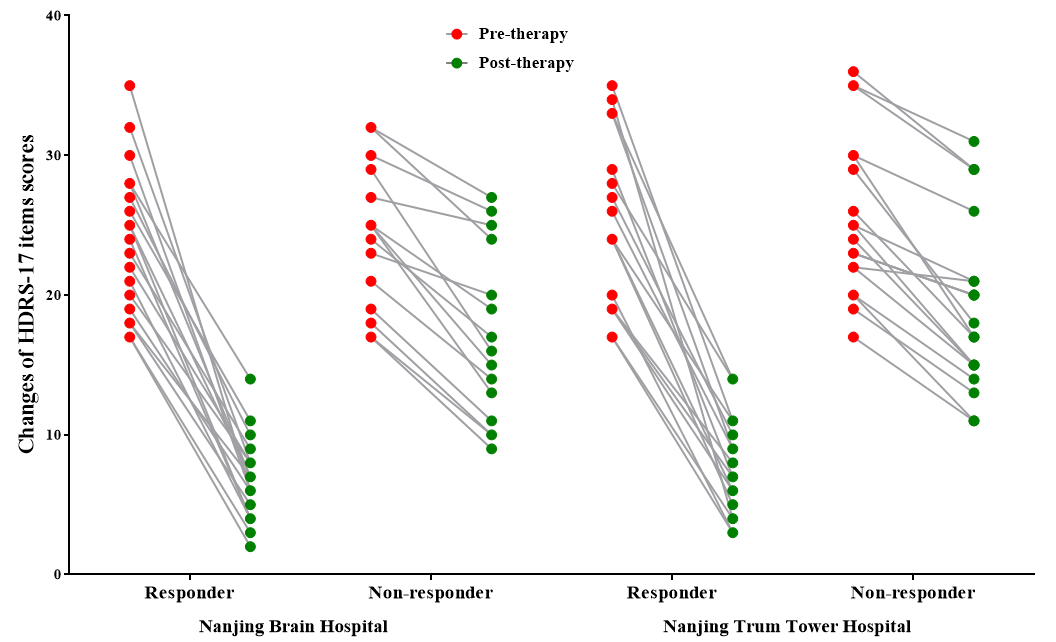


FigS1 The changes of depression severity in MDD patients following treatment


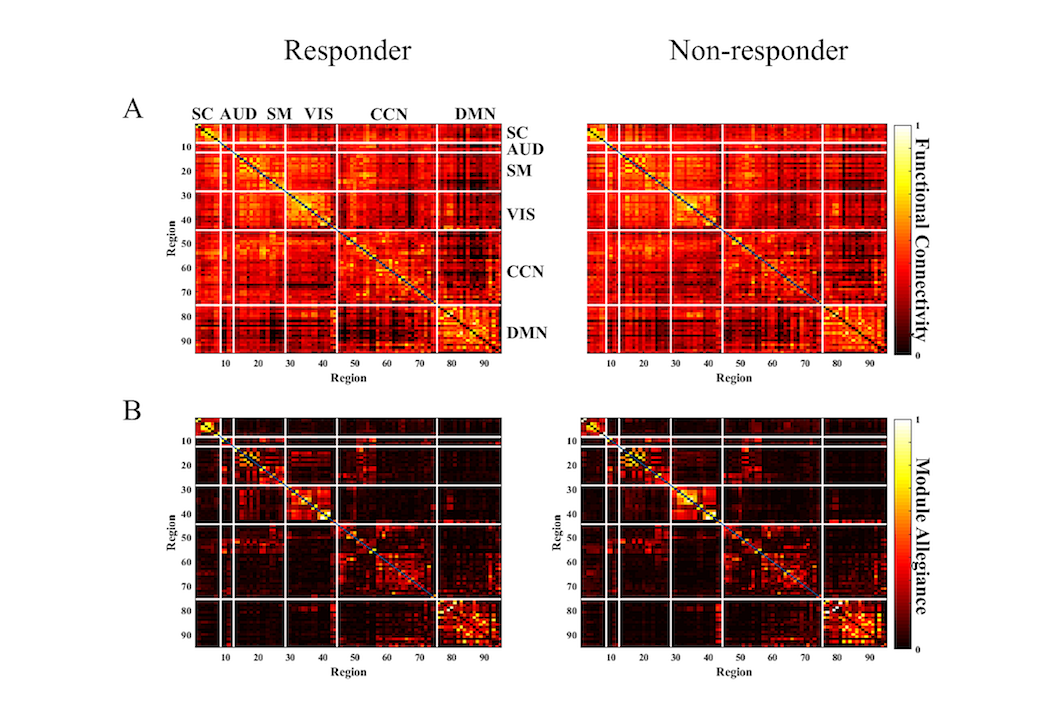


FigS2 Dynamic brain architecture for responders and non-responders of Nanjing Drum Tower Hospital

(A) The averaged functional connectivity matrices for responders and non-responders. The elements correspond to the averaged Pearson correlation between pairs of regions across all responders (B) The averaged module allegiance matrices for two groups. The elements represent the probability of areas that are in the same community across time windows and subjects. The functional architectures were better delineated for intrinsic brain networks. SC: subcortical network; AUD: Auditory network; SMN: somatomotor network; VIS: visual system; CCN: cognitive control network; DMN: Default mode network.


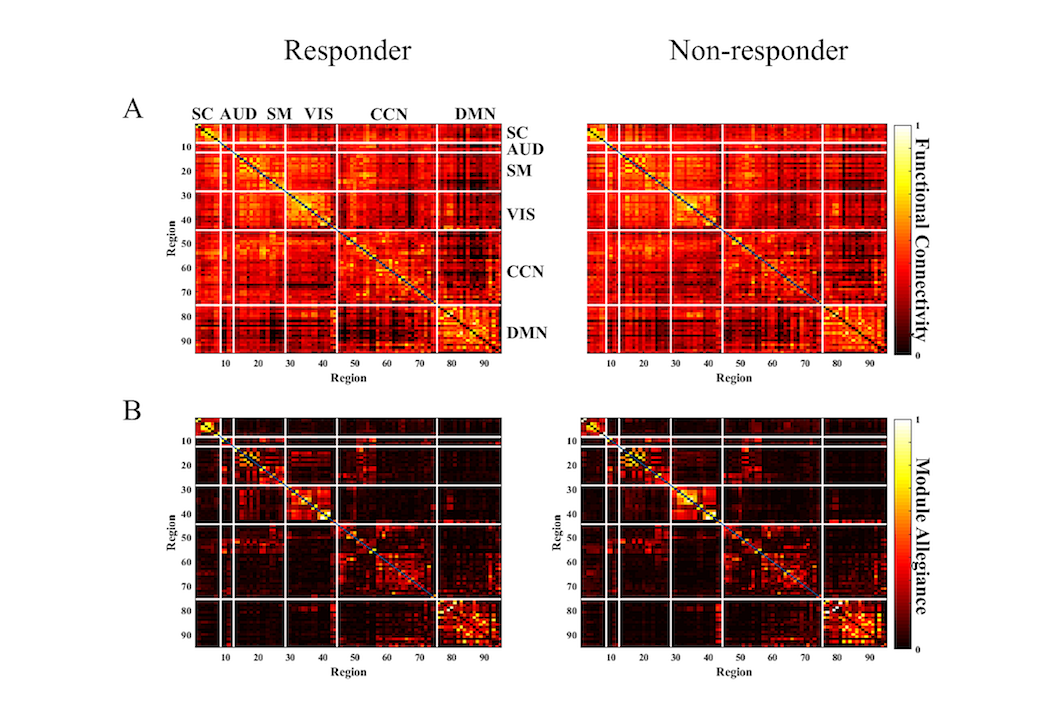


FigS3 Dynamic brain architecture for responders and non-responders of Peking University Institute of Mental Health

(A) The averaged functional connectivity matrices for responders and non-responders. The elements give the averaged Pearson correlation between pairs of regions across all responders (B) The averaged module allegiance matrices for two groups. The elements give the probability of areas that are in the same community across time windows and subjects. The architectures were better delineated for intrinsic brain networks. SC: subcortical network; AUD: Auditory network; SMN: somatomotor network; VIS: visual system; CCN: cognitive control network; DMN: Default mode network.


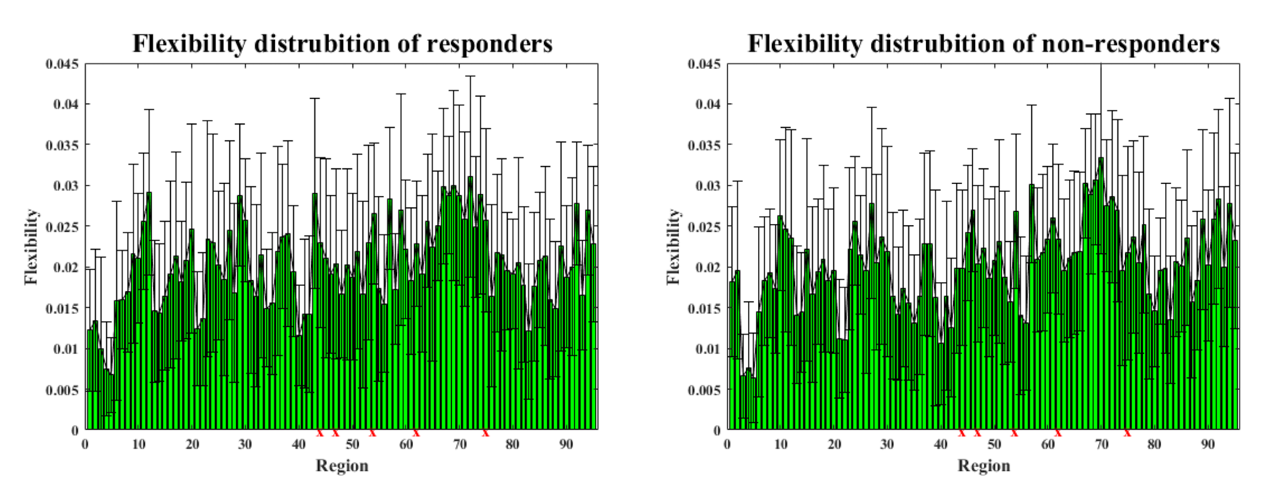


FigS4 Flexibility distribution

Regional flexibilities of two groups were showed in histograms. The horizontal axis represents regions, while the vertical axis represents flexibility distribution described by mean ± a standard deviation. Two samples t-test showed there were significant difference in flexibility of some regions, namely, left superior occipital gyrus(*t*=2.431, *p*=0.021, uncorrected, confidence interval 95% 0.0015-0.0169), right middle frontal gyrus(*t*=-2.668, *p*=0.012, uncorrected, confidence interval 95% -0.0138­- -0.0186), left supplementary motor area (*t*=2.166, *p*=0.038, uncorrected, confidence interval 95% 0.0004- 0.0141), left middle frontal gyrus (*t*=-2.459, *p*=0.020, uncorrected, confidence interval 95% -0.0140- -0.0013) and right para-hippocampal gyrus (*t*=2.260, *p*=0.031, uncorrected, confidence interval 95% 0.0009- 0.0176). These regions were color by red ‘X’ in the figure. In the main text, the features (Fig.2 D) selected using minimum redundancy maximum relevance (mRMR) were major overlapped by these regions.


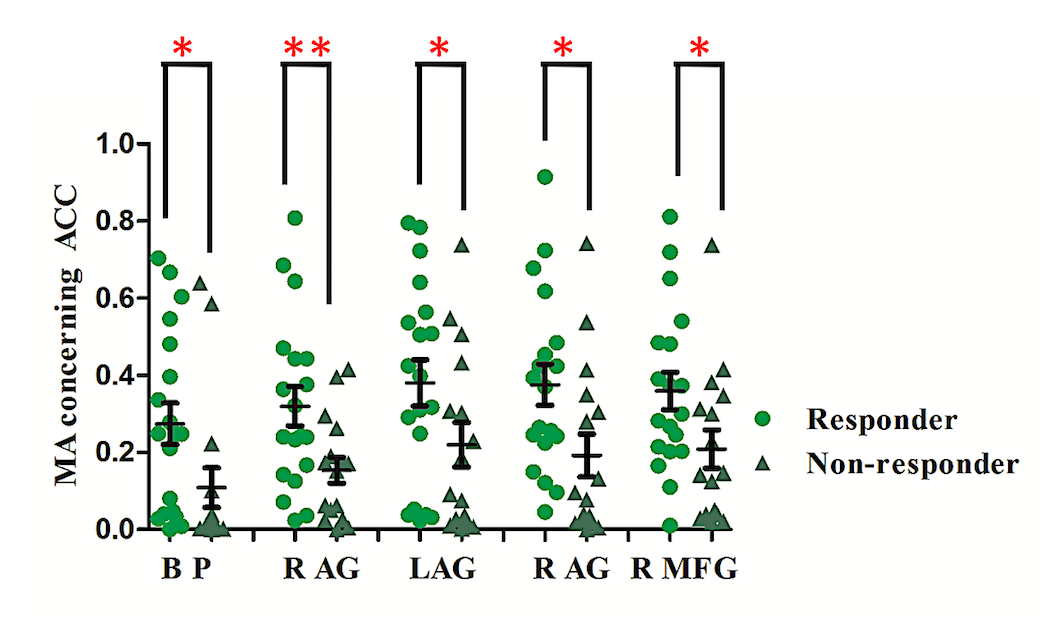


FigS5 MA between ACC to some special regions for samples of Peking University Institute of Mental Health

Scatter plot of MA involving ACC with some special regions and lined by mean ± standard error of the mean, including B P (*t*=2.678, *p*=0.012, uncorrected, confidence interval 95% 0.0475-0.3495), R AG (*t*=3.140, *p*=0.004, uncorrected, confidence interval 95% 0.0676-0.3175), L AG (*t*=3.140, *p*=0.004, uncorrected, confidence interval 95% 0.0094-0.3743), R AG (*t*=2.846, *p*=0.008, uncorrected, confidence interval 95% 0.0608-0.3671) and R MFG (*t*=2.238, *p*=0.032, uncorrected, confidence interval 95% 0.0143-0.3041). A single asterisk indicated p <0.05, two asterisks indicate p <0.005. Bars indicate mean values, and whiskers represent standard error of the mean (SEMs). ACC: bilateral anterior cingulate cortex; B P: bilateral precuneus; R AG: right angular gyrus; L AG: left angular gyrus; R MFG: right middle frontal gyrus.


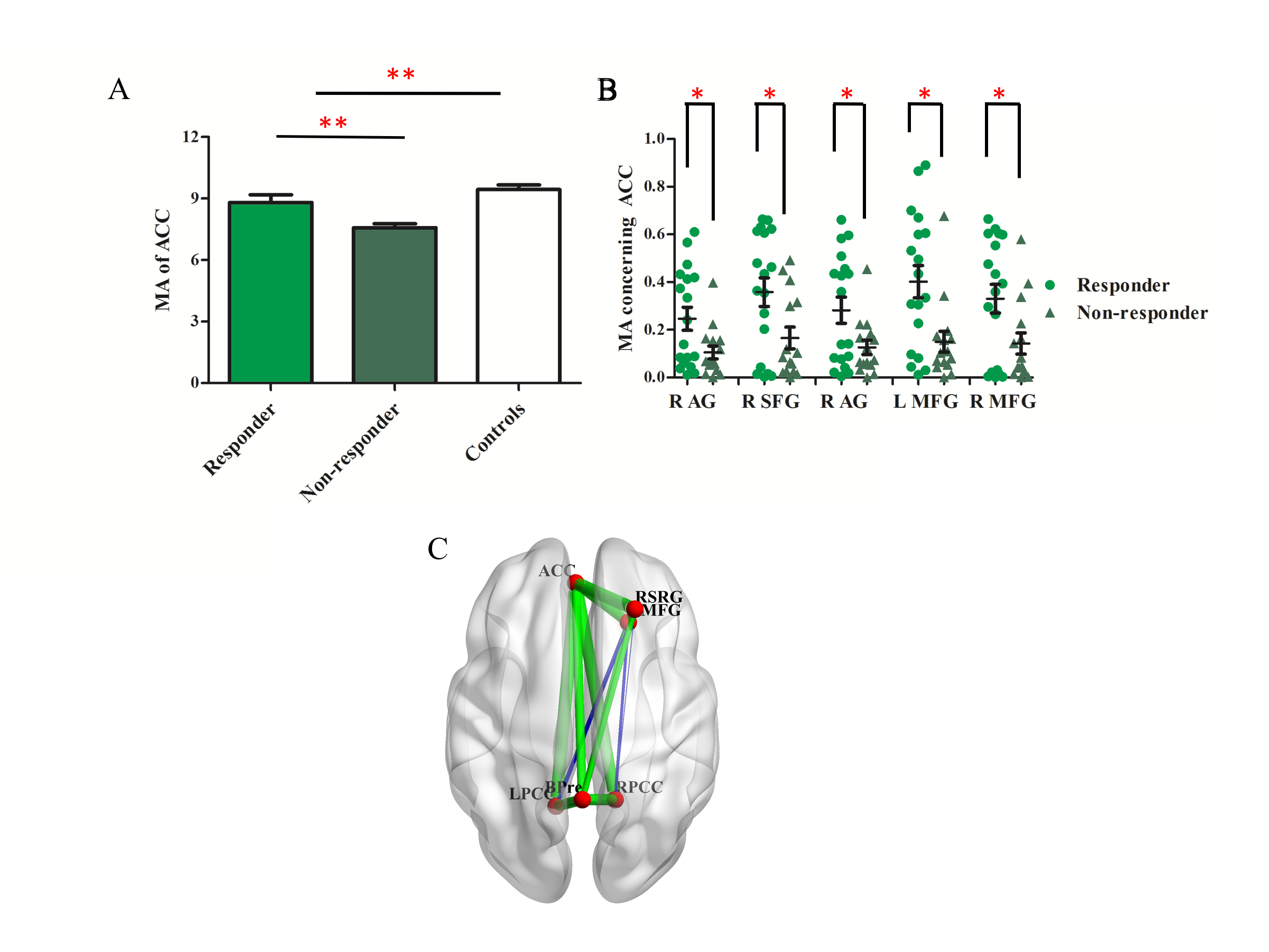


FigS6 MA of the key regions relating to the ACC for samples of Nanjing Drum Tower Hospital

(A) The histogram illustrated the MA between ACC and other brain regions in responders, non-responders and healthy controls. Patients with depression showed lower MA than healthy controls while responders possessed larger one than non-responders. Bars indicate mean values, and whiskers represent standard deviations (SDs). (B) The scatter plot of MA concerning the ACC to some special regions, including R AG, R SFG, L MFG and R MFG. Bars indicate mean values, and whiskers represent SEMs. (C) The difference of MA in the anterior default mode subnetwork between responders and non-responders. A single asterisk indicated p <0.05 and two asterisks indicate p <0.005. Key nodes are shown in red color. Green lines represent positive difference and blue lines represent negative difference. The larger the difference between them, the stronger the line. ACC: bilateral anterior cingulate cortex; B Pre: bilateral precuneus; L PCC: left posterior cingulate cortex; R PCC: right posterior cingulate cortex; R SFG: right superior frontal gyrus R MFG: right middle frontal gyrus.
